# Novel Models for Chronic Intestinal Inflammation in Chickens: Intestinal Inflammation Pattern and Biomarkers

**DOI:** 10.3389/fimmu.2021.676628

**Published:** 2021-05-12

**Authors:** Gabriela C. Dal Pont, Bruna L. Belote, Annah Lee, Cristiano Bortoluzzi, Cinthia Eyng, Milena Sevastiyanova, Alireza Khadem, Elizabeth Santin, Yuhua Z. Farnell, Christos Gougoulias, Michael H. Kogut

**Affiliations:** ^1^ Department of Poultry Science, Texas A&M Agrilife Research, Texas A&M University, College Station, TX, United States; ^2^ Department of Veterinary Science, Federal University of Paraná, Curitiba, Brazil; ^3^ Department of Animal Science, Western Parana State University, Marechal C. Rondon, Brazil; ^4^ Innovad NV/SA, Essen, Belgium; ^5^ Southern Plains Agricultural Research Center, United States Department of Agriculture - Agricultural Research Service (USDA-ARS), College Station, TX, United States

**Keywords:** calprotectin, DSS, gut health, lipocalin, models of intestinal inflammation, ISI index

## Abstract

For poultry producers, chronic low-grade intestinal inflammation has a negative impact on productivity by impairing nutrient absorption and allocation of nutrients for growth. Understanding the triggers of chronic intestinal inflammation and developing a non-invasive measurement is crucial to managing gut health in poultry. In this study, we developed two novel models of low-grade chronic intestinal inflammation in broiler chickens: a chemical model using dextran sodium sulfate (DSS) and a dietary model using a high non-starch polysaccharide diet (NSP). Further, we evaluated the potential of several proteins as biomarkers of gut inflammation. For these experiments, the chemical induction of inflammation consisted of two 5-day cycles of oral gavage of either 0.25mg DSS/ml or 0.35mg DSS/ml; whereas the NSP diet (30% rice bran) was fed throughout the experiment. At four times (14, 22, 28 and 36-d post-hatch), necropsies were performed to collect intestinal samples for histology, and feces and serum for biomarkers quantification. Neither DSS nor NSP treatments affected feed intake or livability. NSP-fed birds exhibited intestinal inflammation through 14-d, which stabilized by 36-d. On the other hand, the cyclic DSS-treatment produced inflammation throughout the entire experimental period. Histological examination of the intestine revealed that the inflammation induced by both models exhibited similar spatial and temporal patterns with the duodenum and jejunum affected early (at 14-d) whereas the ileum was compromised by 28-d. Calprotectin (CALP) was the only serum protein found to be increased due to inflammation. However, fecal CALP and Lipocalin-2 (LCN-2) concentrations were significantly greater in the induced inflammation groups at 28-d. This experiment demonstrated for the first time, two *in vivo* models of chronic gut inflammation in chickens, a DSS and a nutritional NSP protocols. Based on these models we observed that intestinal inflammation begins in the upper segments of small intestine and moved to the lower region over time. In the searching for a fecal biomarker for intestinal inflammation, LCN-2 showed promising results. More importantly, calprotectin has a great potential as a novel biomarker for poultry measured both in serum and feces.

## Introduction

Antibiotics used as growth promoters (AGP) have successfully controlled dysbiosis and enteropathogens for the past 50 years (1). However, the recent increase in worldwide non-AGP poultry production is challenging the industry in management, health, and animal welfare due to the increase of enteric and systemic diseases (2, 3). One of the most accepted theories of AGP mechanism is its role in reducing low-level inflammation (4) and immunologic stress (5) that can be caused by environmental factors, pathogens, and feed ingredients (6). Therefore, removing AGP may increase the inflammatory level of the non-AGP flocks and reduce their performance, which raises the importance of understanding intestinal health and applied practices to its promotion (7).

Despite the importance of studying chronic gut inflammation (8), there is a lack of a practical model to induce a low-grade chronic response in broilers that would mimic field situations. Among the developed models in poultry species, most produce strong negative effects causing clinical signs such as the reduction in body weight (9–13). Other models are difficult to replicate or apply in large experiments due to complex methodology (14), including chemicals furnished *via* drinking water (10), injections (11, 13), or pathogen-specific challenge. Therefore, there is a need for an easily replicated research model that will best mimic the intestinal response in the field.

A nutritional model to produce gut inflammation would be advantageous because the diet would induce a non-specific immune response, also it is easier to apply and might resembles industry situations. Rice bran is an alternative ingredient for energy used in animal nutrition. Compared to other alternatives, such as wheat, rice has higher metabolizable energy and lower fiber content (15). Although rice bran is not considered a soluble ingredient (16), it still has a considerable percentage of fiber, one of the highest phytate content in vegetable ingredients (1.37%), and a large amount of lipids (14.2%) that may suffer peroxidation (15, 17). The negative effects of rancid lipids and phytate on the intestinal mucosa are well known (6, 18–21). Thus, diets with rice bran inclusion may provoke an intestinal challenge in which the degree will vary with the inclusion rate of the ingredient. Alternatively, chemical models of intestinal inflammation also produce a non-specific response that can be used as a positive control in certain situations, although they may not be identical to industry situations and should be used with caution. Dextran sodium sulfate (DSS) is a polymer widely used in rodent studies to produce colitis. DSS has previously been shown to induce intestinal inflammation in chickens. Oral gavage of DSS had been shown to induce loose stools, histology alterations and weight loss (9, 10, 22). However, most of the rodents and all the DSS chicken protocols produced an acute inflammatory response in the intestine. To study chronic intestinal disease, such as Crohn´s and inflammatory bowel diseases, Bento and collaborators (23) developed a protocol that produced chronic intestinal inflammation after subjecting mice through to two cycles of DSS *via* oral gavage.

In addition to establishing a chronic inflammation model, the poultry industry seeks reliable non-invasive biomarker(s) of intestinal inflammation. Several potential biomarkers have already been evaluated in the chicken, such as lipocalin (LCN2), alpha-1 antitrypsin, intestinal alkaline phosphatase, superoxide dismutase, fibronectin (13), and ovotransferrin (24, 25). However, only a few (ovotransferrin, lipocalin and fibronectin) have shown promising but inconsistent results in previous research. Studies in human medicine have proven that fecal concentration of calprotectin, a protein present in neutrophils, monocytes, and macrophages, is a non-invasive method to evaluate the gut inflammation process (26, 27). Recently, calprotectin has been used to aid diagnosis of colonic diseases, for monitoring disease and cancer, and to evaluate efficacy of treatments (28–31). To our knowledge, there have been no studies quantifying levels of calprotectin in poultry excreta or fluids. However, increased gene expression of calprotectin was identified in the intestine of broilers exposed to *Salmonella enterica* (32), *Eimeria maxima* and *Clostridium perfringens* (33), and heat stress (34). Thus, we believe calprotectin has the potential to serve as a biomarker of inflammation in the small intestine of broilers.

Therefore, for this study, we hypothesized that a diet with high inclusion of rice bran or a challenge with DSS based on the Bento et al. (33) protocol have the potential to induce a low-grade chronic inflammation in the intestine of chickens. In the current study, we utilized a) a diet with 30% of rice bran and b) two cycles of DSS oral administration at two different levels, to produce a model of low-grade intestinal inflammation in broilers. Moreover, we evaluated the effect of the challenges overtime in the small intestine and analyzed several biomarkers in the blood and excreta of chickens. This allowed us to: (1) identify two simplistic low-grade inflammation models without inducing major clinical signs and/or a pathogenic infection (2), evaluate calprotectin as a novel biomarker, and (3) quantify in low-grade chronic inflammation model potential biomarkers previously reported.

## Material and Methods

The experiment was conducted in accordance with guidelines set by the United States Department of Agriculture Animal Care and Use Committee (USDA IACUC #2019012). The trial was conducted at the Agricultural Research Service Facility of the United States Department of Agriculture (ARS-USDA), College Station, Texas, US.

**Figure 1:**
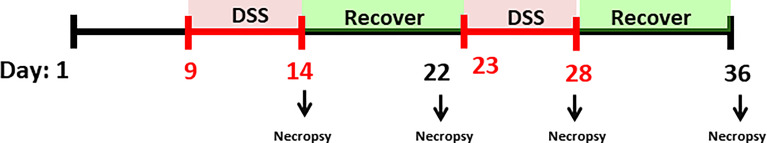
Experimental timeline. Male by-product day-of-hatch chickens were divided in four experimental treatments and raised up to 36 days. The broilers assigned to treatments with DSS challenge received 0.25mg/ml or 0.35mg/ml of DSS *via* oral gavage everyday from 9 to 14-d and 23 to 27-d (induction phase period represented with red). After each DSS cycle animals were allowed to a 9 days recovery period (from 14 to 23-d and 27 to 36-d, marked in green). Birds in the NSP treatment received a diet with 30% of rice bran from 1 to 36-d. Animals in the control group were not submitted to any challenge. Necropsies were performed on days 14, 22, 28 and 36 for collection of intestinal tissue, blood, and fecal samples.

To evaluate the two different intestinal inflammation models, a total of 180 Cobb male by-product day-of-hatch chickens were randomly assigned to four experimental treatment groups with three repetitions of 15 birds each. The treatments were: (1) Control (CNT) with a corn/soybean meal standard diet; the second and third groups received control diet and were submitted to two cycles of (2) 0.25mg/ml of DSS (25DSS), or (3) 0.35mg/ml of DSS (35DSS), respectively; and (4) the fourth group had a high non-starch polysaccharide diet (NSP) (30% of rice bran) (**Table 1**). DSS was used as a chemical inducer of intestinal inflammation. The birds in 25DSS- and 35DSS-treated groups received a DSS solution administrated *via* oral gavage in two cycles of 5 consecutive days, interrupted by a recovery period of 9 days as described for a murine model of intestinal chronic inflammation (23). The first cycle lasted from day 9 to day 14 of age; the second cycle was applied from day 23 to day 27 of age (**Figure 1**). Birds in the NSP treatment received feed with 30% of rice bran inclusion during the entire experimental period (1 to 36-d of age).

**Table 1:** Experimental treatments.

Treatment	Abbreviation	Diet	Intestinal Challenge
Control	CNT	Corn/soybean meal standard diet	No
0.25mg/ml of DSS	25DSS	Corn/soybean meal standard diet	0.25mg/ml of DSS oral gavage daily from 9 to14-d and 23 to 27-d
0.35mg/ml of DSS	35DSS	Corn/soybean meal standard diet	0.35mg/ml of DSS oral gavage daily from 9 to14-d and 23 to 27-d
High NSP	NSP	30% of rice bran inclusion	High NSP diet

NSP, non-starch polyssacharide; CNT, Control; DSS, Dextran sodium sulfate.

The different diets offered were iso-energetic, iso-nitrogenous, and formulated to meet or exceed the broiler’s requirements (Cobb manual, 2018) (**Table 2**). Two feeding phases were used: starter (1-21 days) and grower (21-36 days). Dextran sodium sulfate solutions were produced by solubilizing DSS (MW ca 40,000; Sigma Aldrich) in distilled water to the desired concentrations.

**Table 2:** Ingredients and calculated nutritional composition of experimental diets.

Ingredients (%)	Starter (1-21 d)		Grower (22-36 d)	
	Control	High NSP	Control	High NSP
Corn	58.87	31.08	65.97	38.185
Soybean meal^1^	34.75	29.93	27.87	22.515
Rice bran	0	30	0	30
Soybean oil	2.40	5.62	2.74	5.95
Monocalcium phosphate	1.72	1.333	1.42	1.03
Limestone	1.08	1.255	0.93	1.10
NaCl	0.37	0.35	0.37	0.35
DL-Methionine	0.35	0.35	0.27	0.30
L-Lysine HCl	0.22	0.29	0.18	0.259
L-Threonine	0.098	0.165	0.012	0.079
Choline chloride	0.05	0.05	0.05	0.05
Vitamin premix^2^	0.05	0.05	0.1	0.1
Mineral premix^3^	0.03	0.03	0.05	0.05
**Calculated composition**				
Metabolizable energy (kcal/kg)	2990	2990	3100	3100
Crude protein (%)	22	22	19	19
Lysine dig. (%)	1.22	1.22	1.02	1.02
Methionine (%)	0.61	0.63	0.53	0.55
Meth. + cysteine dig. (%)	0.91	0.91	0.80	0.80
Threonine dig. (%)	0.83	0.83	0.66	0.66
Av. Phosphorus (%)	0.45	0.45	0.38	0.38
Calcium (%)	0.90	0.90	0.76	0.76
Potassium (%)	0.92	1.14	0.79	1.02
Sodium (%)	0.16	0.16	0.16	0.16

^1^ Soybean meal 49% of crude protein.

^2^ Composition of minimum (per kg of feed): Vit A 8,818,342 IU; Vit D3 3,086,420 IU; Vit E 3,674 IU; Vit B12 130 mg; Vit K 1,177mg; Vit B2 4,775 mg; pantothenic acid 16,168 mg; Vit B1 2,350 mg; Vit B3 36,742 mg; Vit B6 5,732 mg; folic acid 1.1,398 mg; choline 104,460 mg; biotin 441mg.

^3^ Minimum of Fe 12%; Cu 1.4%; I 800ppm; Zn 12%; Mn 173.0 mg; Mg 12%.

Av., available; dig, digestible.

Chickens were placed from day 1 to 36 of age on 0.91 x 1.21 m^2^ pens and a minimum of 0.074m^2^/bird was maintained during the whole experimental period; the floor was covered with new pine shavings. Water and feed were offered *ad libitum* and environmental conditions were maintained for each growing phase according to the Cobb manual recommendations (2018).

### Necropsy and Sampling

Necropsies were performed on days 14, 22, 28 and 36 of age and intestinal tissue, blood, and fecal samples were collected to evaluate the gastrointestinal tract (GIT) inflammation (**Figure 1**). At necropsy, two birds from each pen (6 birds from each treatment) were randomly selected for sample collection. Initially, blood was collected in serum tubes from the jugular vein, and finally the bird was euthanized by cervical dislocation. The collected blood tubs were laid flat for clotting (at least for 30 min) and the serum was separated and collected after centrifugation (15 min on 3000 rpm) and stored at -20°C for further analyses.

Following euthanasia, each carcass was systematically evaluated to identify the health status and physical condition of the gastrointestinal tract based on the “I See Inside” (ISI) methodology (35). The intestine (duodenum, jejunum, ileum and cecum) and GIT-associated organs (liver, yolk, proventriculus, gizzard, pancreas) were evaluated grossly as described (35). Several macroscopic characteristics were scored on a 0-3 scale and the scores were then multiplied by impact factors (1–3), depending on their importance (in detail in **Table 1** of “**Supplemental Data**”). The ISI macro total score (sum of all observed lesions) in the study can reach 78, stemming from a total intestinal score of 54 and a total GIT-associated organs of 24. The higher the ISI score, the worse the GIT health status.

During the necropsy, samples of duodenum, jejunum and ileum were collected for posterior histological analysis. For the tissue collection, intestinal anatomy was used to ensure sampling of a similar segment in all birds. Duodenum samples (5cm) were taken 1cm caudal the duodenal loop. Meckel´s diverticulum was used to separate jejunum and ileum and the sampling was made in the middle of each segment (5cm). The intestinal samples were washed with 0.9% NaCl solution and fixed in 10% buffered formalin.

### Fecal Sampling

Fresh excreta samples were collected at 28 and 36-d from 2 birds per repetition (6 samples for each treatment), immediately frozen in liquid nitrogen and stored at -80°C. For downstream ELISA array, 0.4 g of excreta was vortexed with 4 ml of PBS and centrifuged for 20 min at 4°C at 3000 rpm. The supernatant was removed and stored at -20°C for posterior assays.

### ELISA Analysis

For the biomarker searching concentration of calprotectin (avian MRP-126), lipocalin, S100 protein, hypoxia inducible factor- 1 subunit alpha (HIF1α), lipopolysaccharides (LPS) and ovotransferrin (OVT) were quantified using the fecal homogenate and/or serum at different time points (**Table 3**). All biomarkers were quantified with ELISA kits from MyBiosource Inc. (San Diego, CA-USA).

**Table 3:** Proteins quantified in serum and/or feces of broilers at 14, 22, 28 or 36 days post-hatch by ELISA.

	14-d	22-d	28-d	36-d	ELISA catalog #
Calprotectin (avian MRP126)	Serum	Serum	Feces	Feces	MBS1601938
Lipocalin	Serum	Serum	Serum and Feces	–	MBS005459
S100A	–	–	Feces	Feces	MBS2504874
HIF1α	–	–	Feces	Feces	MBS287105
Ovotransferrin	–	–	Feces	–	MBS2533639
LPS	Serum		Serum	Serum	MBS268415

S100A, S100A protein; HIF1α, hypoxia inducible factor-1 subunit alpha; LPS, lipopolysaccharides.

### Histology and I See Inside Methodology

The intestinal samples stored in formalin were dehydrated, infiltrated, and embedded in paraffin following standard histological practices. Paraffin blocks were cut into 5 μm sections and stained with hematoxylin and eosin.

For evaluation of microscopic alterations, 20 villi sections per bird were evaluated at 10X magnification (using 40X magnification to confirm alterations) by a treatment blinded person using an optical microscope (Nikon Eclipse E200, Sao Paulo, Brazil). The “I See Inside” (ISI) microscopy methodology (patent INPI BR 1020150036019), was used to measure histologic alterations on the intestine (36), and a final intestinal health index was produced. A score (0–3) was given to each alteration depending on the extent of the lesion: the greater the lesion, the higher the score. The score was then multiplied by an impact factor (IF; 1-3) which is based on the consequence of alteration on the organ functionality. The final ISI index was then calculated as the sum of all alteration index:

$$ISI\;index=\;\sum^{\;}score*IF$$

The alterations evaluated, and their impact factor were: lamina propria thickness*(2), epithelial thickness*(1), enterocytes proliferation*(1), inflammatory cell infiltration in the epithelium*(1), inflammatory cell infiltration in the lamina propria*(3), goblet cells proliferation*(2), congestion*(2), and the presence of *Eimeria sp*. oocysts*(3). Therefore, animals with a high final ISI index had worse intestinal health. For statistical analysis each villus was considered as a replicate.

### Statistical Analysis

Data were analyzed accordingly to a complete randomized design. At first, normality of all the data was verified through Shapiro-Wilk’s test and variables with non-normal distribution were analyzed by Kruskal Wallis test, as all data generated by the microscopic and macroscopic ISI evaluation. Then, data with normal distribution were analyzed using ANOVA (P-value <0.05), and means were compared by Tukey. Dunnet’s test was also used in the biomarkers data to compare challenged groups with the CNT group. For feed intake, each pen was used as an experimental unit, and for the remaining analyses, each bird was used as an experimental unit. Due to the low number of experimental units (3 pens per treatment), feed intake was only used as a clinical sign and not to compare performance between groups. Software JMP^®^ Pro 15.0.0 (SAS Institute Inc.) was used, and for all tests P-value <0.05 was considered statistically significant.

## Results

### Chronic Inflammation Model

#### Macroscopic Gut Observations

It was observed that neither the DSS treatment nor the rice bran diet had an effect on feed intake or mortality (“**Supplemental Data**”- **Table 2**). However, necropsy revealed intestinal lesions in birds treated with DSS or fed the rice bran diet (**Table 4**). By the macroscopic “I See Inside” method, NSP exhibited a more consistent pattern of inflammation which negatively affected the intestinal health as seen by the significantly higher macroscopic lesion scores on days 14 and 36 with a similar trend on days 22 and 28 (**Table 4**). Both DSS cycles and the high NSP diet induced localized intestinal lesions, but no gross inflammatory lesions in extra-intestinal organs (pancreas, yolk-sac, liver, gizzard and proventriculus) were observed. Moreover, the macroscopic ISI score lesions of each section of the intestine showed that the duodenum was affected more in the early days of the treatments (**Table 5**) whereas, the posterior parts of the intestine showed compromised intestinal health at a later stage (36 days), suggesting a spatial and temporal impact on the intestinal lumen.

**Table 4:** Macroscopically I See Inside (ISI) lesions scores of related organs, intestine and total score of broilers submitted to different intestinal challenges at 14, 22, 28 and 36 days of age. The broilers challenged with DSS received 0.25mg/ml (25DSS) or 0.35mg/ml (35DSS) of DSS via oral gavage everyday from 9 to 14-d and 23 to 27-d; birds in the NSP treatment received a diet with 30% of rice bran during the whole experiment, and animals in the control group were not submitted to any challenge.

Treatment	14 days			22 days			28 days			36 days		
	Related organs	intestine	Total	Related organs	Intestine	Total	Related organs	Intestine	Total	Related organs	Intestine	Total
Control	4.83	5.66 b	10.50^b^	2.66	5.00	7.66	2.16	5.50	7.66	1.83	1.50 b	3.33
25DSS	4.50	9.33 ab	14.16^ab^	3.33	5.50	8.83	2.33	9.33	11.66	1.00	4.83 ab	5.83
35DSS	5.16	10.66 ab	15.83^ab^	1.83	7.16	9.00	2.66	6.16	8.83	1.83	3.50 b	5.33
NSP	4.83	13.66 a	18.50^a^	3.66	6.33	10.00	2.50	9.66	12.16	0.83	7.83 a	8.66
**SEM^1^**	1.224	1.686	1.804	0.850	1.139	1.285	0.677	1.381	1.770	0.7216	1.0672	1.4912
**P-value**	0.985	0.024	0.036	0.456	0.563	0.651	0.958	0.098	0.241	0.656	0.003	0.122

Related organs evaluated: liver, yolk sac, proventriculus, gizzard, pancreas.

^ab^ Different superscript letters indicate significant difference with Tukey test.

^1^ Pooled standard error of the mean.

Maximum macroscopic ISI score: Related organs 24; Intestine 54; Total 78.

n= 6 animals/treatment; 2 animals/pen.

**Table 5:** Macroscopically I See Inside lesions scores of duodenum (Duod), jejunum (Jejun), ileum and ceca of broilers submitted to different intestinal challenges at 14, 22, 28 and 36 days of age. The broilers challenged with DSS received 0.25mg/ml (25DSS) or 0.35mg/ml (35DSS) of DSS via oral gavage everyday from 9 to 14-d and 23 to 27-d; birds in the NSP treatment received a diet with 30% of rice bran during the whole experiment, and animals in the control group were not submitted to any challenge.

Treatment	14 days				22 days				28 days				36 days			
	Duod	Jejun	Ileum	Ceca	Duod	Jejun	Ileum	Ceca	Duod	Jejun	Ileum	Ceca	Duod	Jejun	Ileum	Ceca
Control	1.5^b^	2.00	1.33	0.83	1.5	0.66	1.50	1.33	1.66	1.50^bc^	1.5	0.83	0.66	0.00 ^b^	0.166^c^	0.66
25DSS	2.66^b^	2.83	1.50	2.33	2.33	0.83	0.66	1.66	1.83	3.50^b^	1.5	2.5	2.00	1.83^ab^	0.5^bc^	0.50
35DSS	3.83^ab^	3.16	1.83	1.83	1.66	2.00	1.33	2.16	1.66	0.66^c^	1.66	2.16	0.66	0.83^b^	1.00^ab^	1.00
NSP	5.66^a^	3.16	2.66	2.16	0.33	3.00	1.50	1.50	1.66	4.00^a^	3.00	1.00	0.83	3.66 ^a^	2.00^a^	1.33
**SEM^1^**	0.8113	0.5809	0.540	0.631	0.667	0.606	0.383	0.524	0.554	0.709	0.621	0.509	0.602	0.747	0.263	0.318
**P-value**	0.0157	0.4338	0.4932	0.3789	0.1891	0.0907	0.3314	0.714	0.9955	0.0164	0.3016	0.0535	0.3481	0.0240	0.0042	0.2881

^ab^ Different superscript letters indicate significant difference with Tukey test.

^1^ Pooled standard error of the mean. n = 6 animals/treatment; 2 animals/pen.

#### Microscopic Histological Gut Observations and Alterations

**Figure 2:**
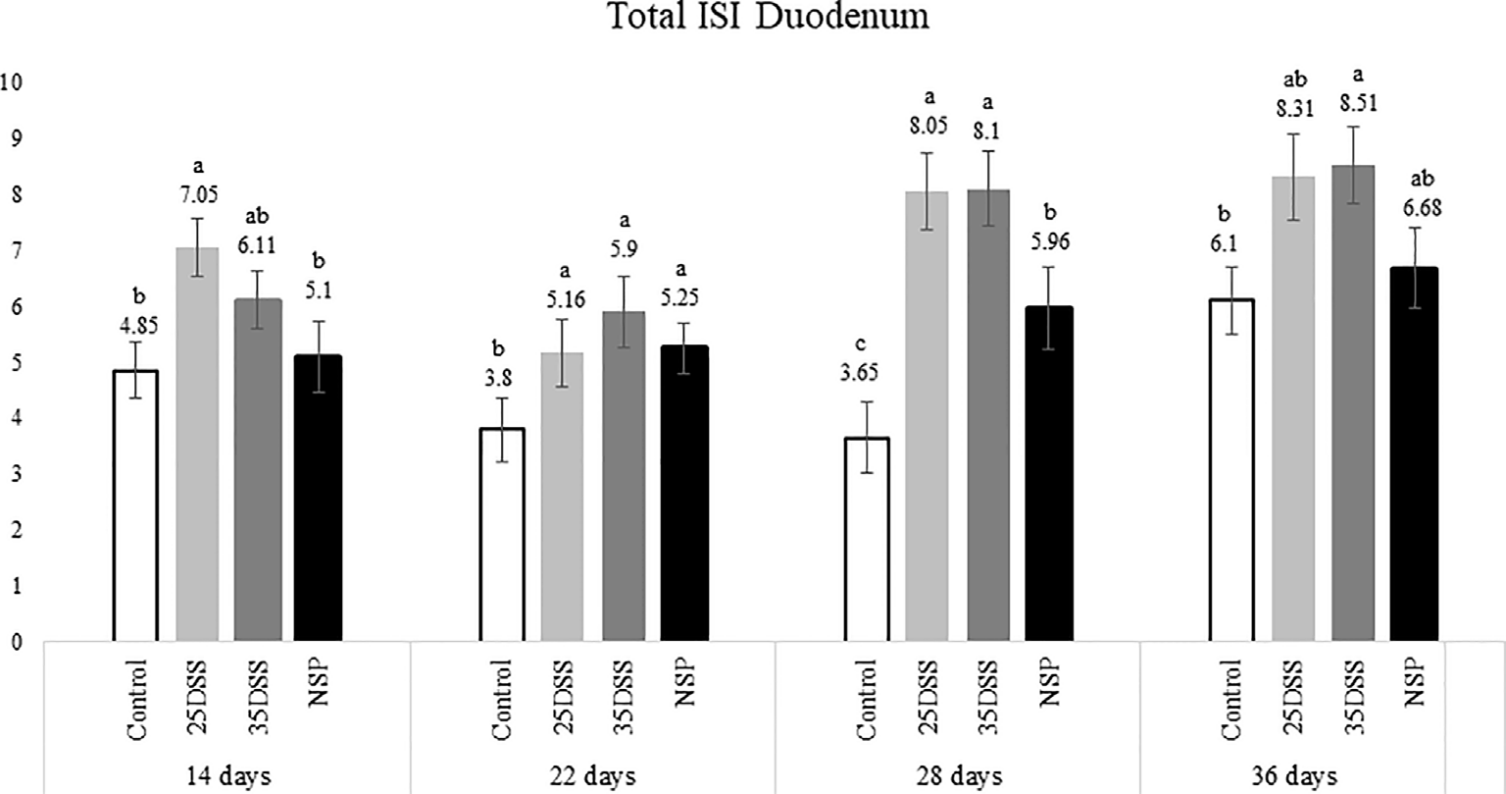
I See Inside (ISI) total microscopically lesions scores of duodenum of broilers submitted to different intestinal challenges at 14, 22, 28 and 36 days of age. The broilers challenged with DSS received 0.25mg/ml (25DSS) or 0.35mg/ml (35DSS) of DSS *via* oral gavage everyday from 9 to 14-d and 23 to 27-d; birds in the NSP treatment received a diet with 30% of rice bran during the whole experiment, and animals in the control group were not submitted to any challenge. ^abc^ Different superscript letters indicate significant difference with Tukey test. n = 6 animals/treatment; 2 animals/pen.

**Figure 3:**
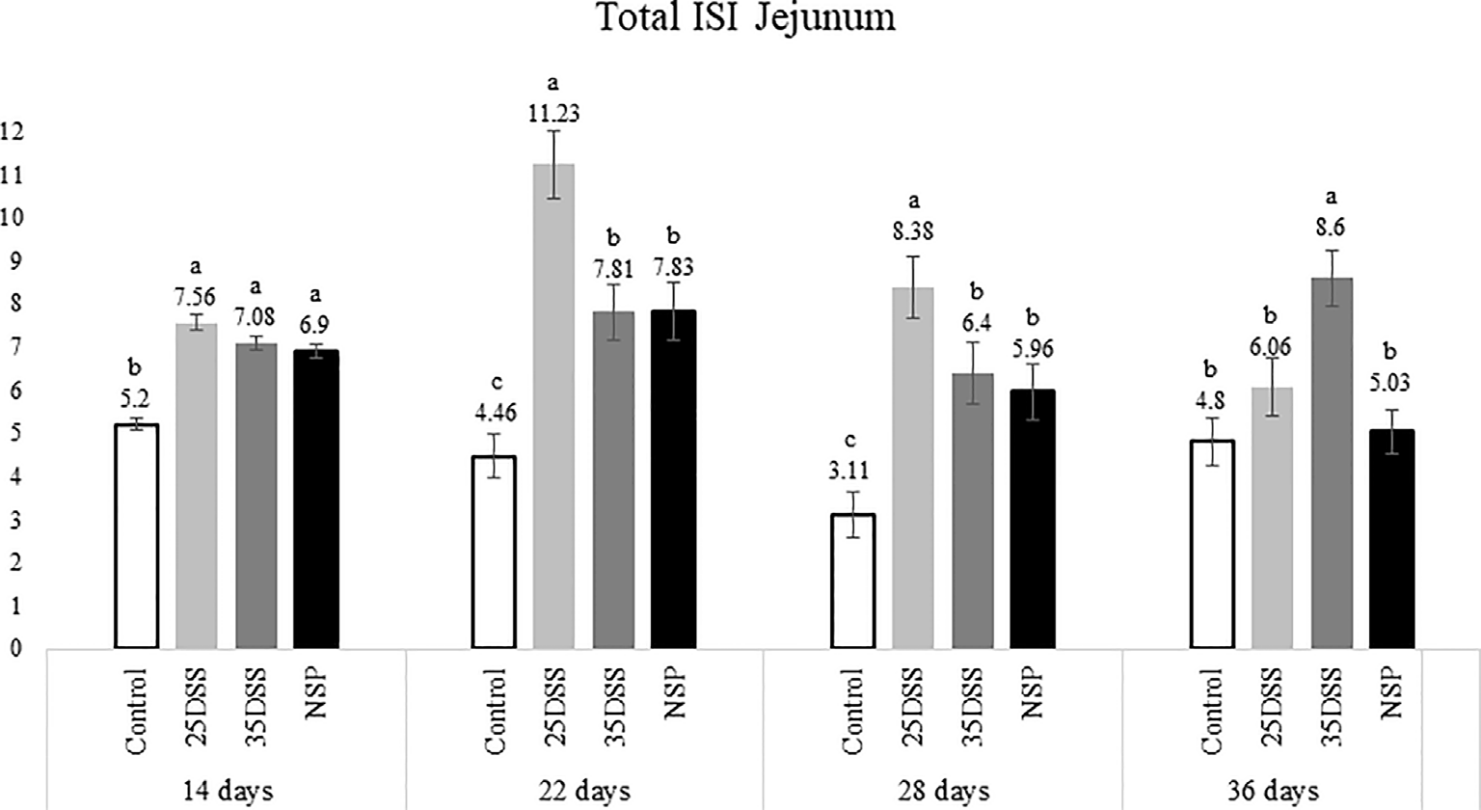
I See Inside (ISI) total microscopically lesions scores of jejunum of broilers submitted to different intestinal challenges at 14, 22, 28 and 36 days of age. The broilers challenged with DSS received 0.25mg/ml (25DSS) or 0.35mg/ml (35DSS) of DSS *via* oral gavage everyday from 9 to 14-d and 23 to 27-d; birds in the NSP treatment received a diet with 30% of rice bran during the whole experiment, and animals in the control group were not submitted to any challenge. ^abc^ Different superscript letters indicate significant difference with Tukey test (P < 0.05). n = 6 animals/treatment; 2 animals/pen.

**Figure 4:**
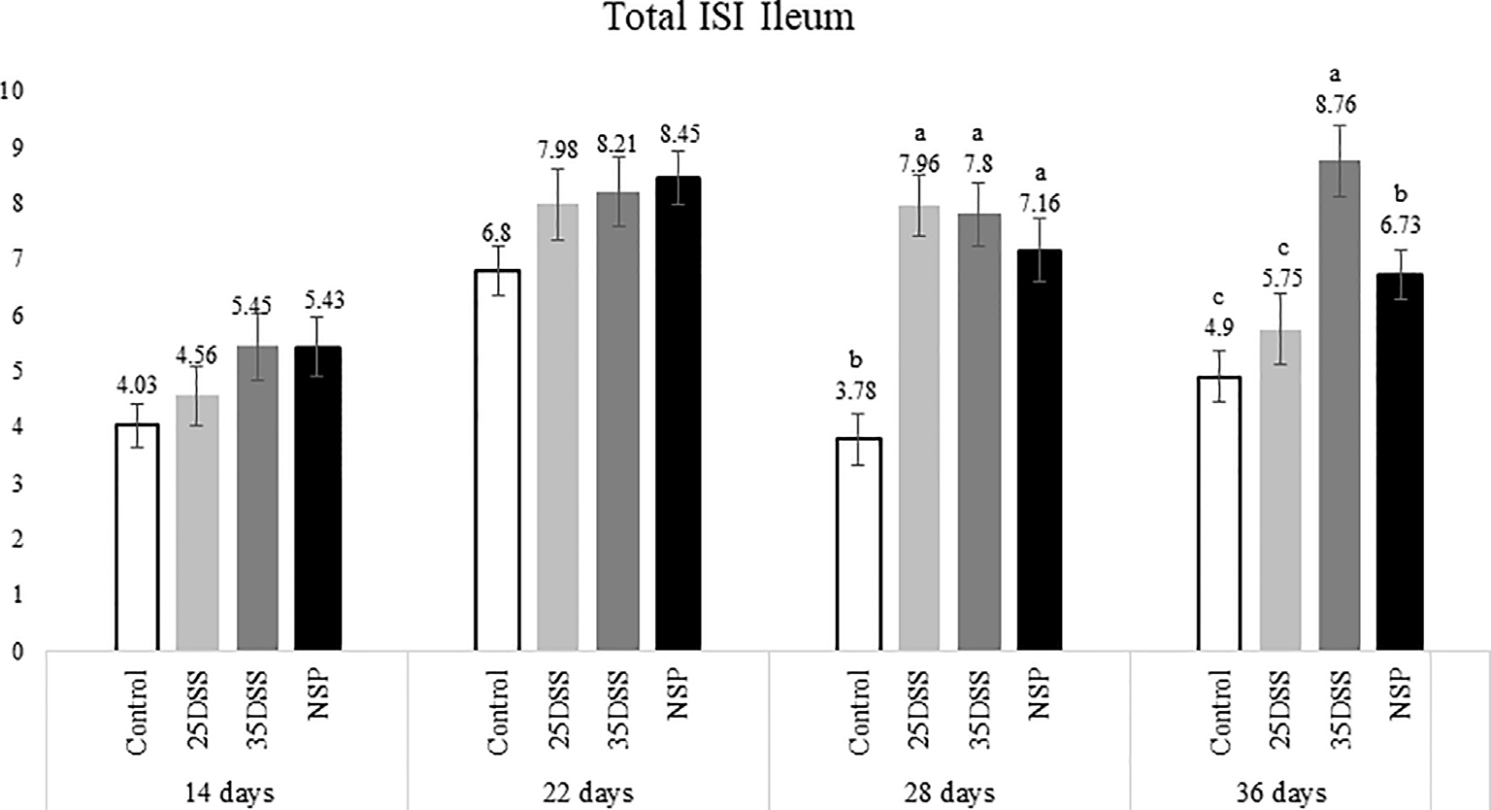
I See Inside (ISI) total microscopically lesions scores of ilea of broilers submitted to different intestinal challenges at 14, 22, 28 and 36 days of age. The broilers challenged with DSS received 0.25mg/ml (25DSS) or 0.35mg/ml (35DSS) of DSS *via* oral gavage everyday from 9 to 14-d and 23 to 27-d; birds in the NSP treatment received a diet with 30% of rice bran during the whole experiment, and animals in the control group were not submitted to any challenge. ^abc^ Different superscript letters indicate significant difference with Tukey test (P < 0.05). n = 6 animals/treatment; 2 animals/pen.

The histological examination of the intestine provided us more detailed and sensitive information about the gut status of the broilers. The microscopic ISI revealed a clear spatial and temporal impact of the treatments in the intestinal lumen. Histologically, the ileum of the birds was affected later, after 28 days, while duodenum and jejunum presented inflammation already in the first necropsy at 14 days of age (**Figures 2**–**4**).

The NSP diet induced higher microscopic ISI scores than the CNT. These difference started to be seeing at 22-d in the duodenum and at 14-d in the jejunum, followed by a stabilization ISI score in these segments by 36-d (**Figures 2** and **3**). However, histological alterations in ileal tissue did not appear until 28-d of NSP diet consumption (**Figure 4**). The main alteration observed in broilers fed the high NSP diet was increased infiltration of inflammatory cells in the lamina propria and epithelium which also seemed to shift in a temporal (from 14-d to 26-d) and spatial fashion (from duodenum towards ileum; **Supplemental Data- Tables 3**–**5**). At later stages of growth (22-d to 36-d), an increase in epithelial and lamina propria thickness was also observed (**Supplemental Data- Tables 3**–**5**). Broilers fed the NSP diet also had a variation in the goblet cells probably as a response to the mucus production by the intestine. Goblet cells were initially (14-d) reduced in the duodenum in this treatment compared to control (P < 0.0001), but at 22 and 28-d of age broilers showed an increase in goblet cells at duodenum and jejunum (P<0.01; **Supplemental Data- Tables 3**, **4**) (**Supplemental Data- Table 5**). This goblet cell response was stabilized at 36 days of age (P < 0.0001; **Supplemental Data- Table 6**). Moreover, birds fed with the high NSP diet presented an increased abnormal proliferation of enterocytes which was constant in the jejunum throughout the experiment, and affected the whole small intestine by 28 days (**Supplemental Data- Tables 3**–**6**).

The chemical model of low-grade inflammation (two cycles of DSS) also was able to reduce intestinal health as indicated by the increase in the ISI histological scores. The duodenal and jejunal tissue were already affected by the first DSS cycle (**Figures 2**, **3**), but the ileum showed a significant increase in ISI only after the second DSS challenge. Even after 9 days of recovery, the duodenum and jejunum had higher ISI scores than the control (**Figures 2**, **3**) and after the second DSS cycle (28-d), the birds presented several histological alterations diffused in the small intestine showing an additive effect of the cycles over the time. The observations of the 28-d are noteworthy because all evaluated parameters were elevated. The duodenum, jejunum and ileum presented higher scores for lamina propria and epithelial thickness, proliferation of enterocytes, and infiltration of inflammatory cells in epithelium and lamina propria than CNT (**Supplemental Data- Table 5**). Moreover, an increase in goblet cells is observed in duodenum and jejunum at that time point.

Lastly, the ISI scores of the 35DSS treatment were higher across all intestinal segments at 36 days showing a decreased intestinal health even after 9-d of recovery from the second DSS cycle. At the end of the experiment, animals in the 35DSS group had higher inflammatory cell infiltration in lamina propria and epithelium as well as a consistent increase in globet cells in all intestine segments (P-value < 0.05), higher lamina propria and epithelial thickness in jejunum and ileum (P-value < 0.01), and increased enterocytes proliferation in the jejunum (P-value = 0.0003; **Supplemental Data**). Thus, the histological alteration indicates that even following 9 days of recovery, two cycles of 0.35mg/ml of DSS were able to induce a long-lasting inflammation.

**Figure 5:**
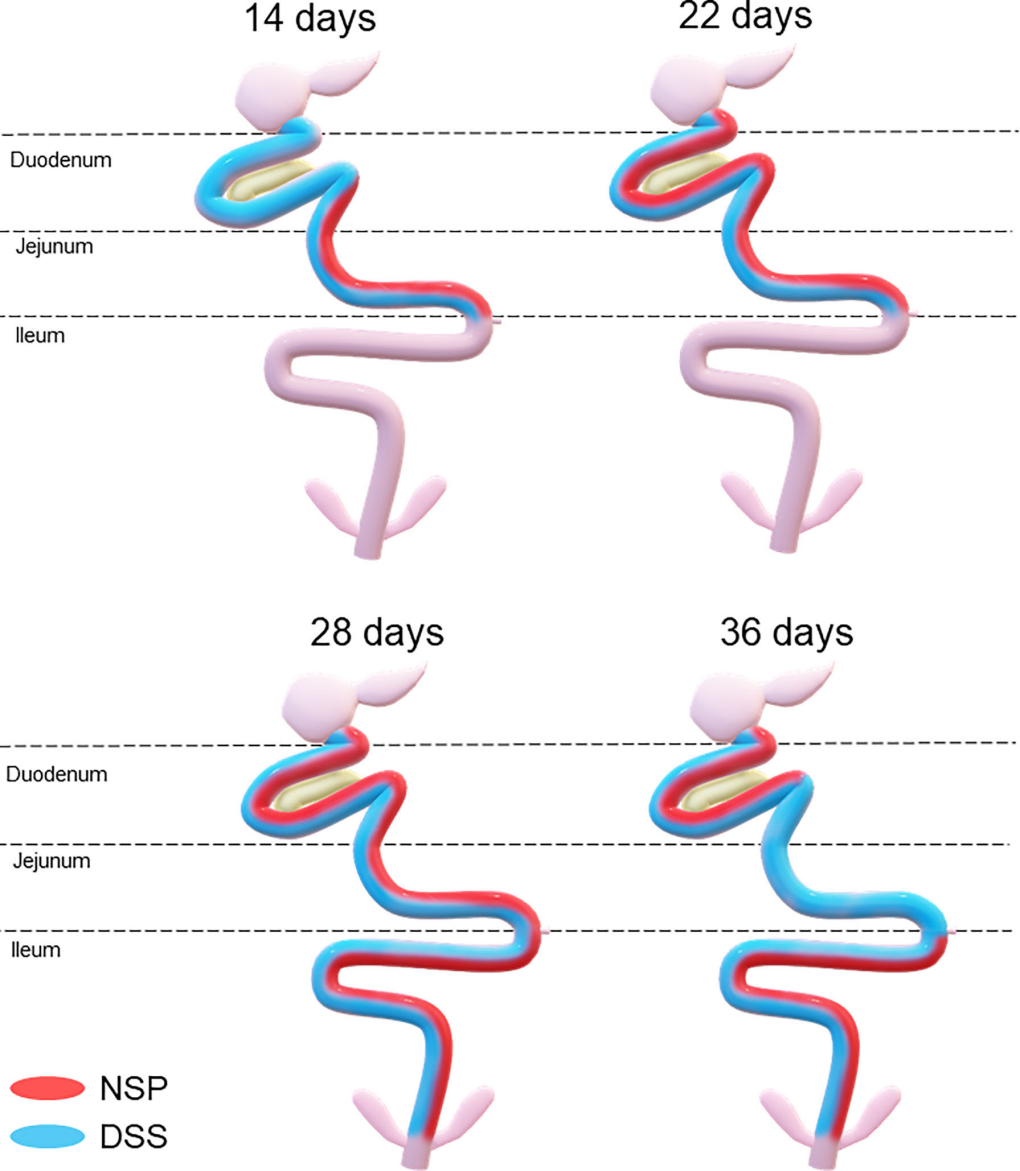
Temporal and spatial evolution of the intestinal inflammation response triggered by a high non-starch pollisacharyde diet (NSP) diet or a DSS challenge in broilers. Areas painted with red and/or blue signalizing the affected* areas in birds fed NSP diet (red) or in birds challenged with the DSS protocol (blue). *affected areas: areas with poor intestinal health (higher microscopic ISI scores).

Therefore, both the high inclusion of rice bran and the two DSS models proved successful in producing chronic intestinal inflammation. The NSP diet induced a marked inflammatory response through the first 28 days of broiler age whereas, the birds showed a partial reduction of the intestinal inflammation when they aged (observed at 36-d). Additionally, the NSP diet produced a lower-grade inflammation when compared to the DSS treatments, the latter presenting consistently a higher ISI score (**Figures 2**–**4**). The two DSS cycles of 0.35mg/ml produced a long-lasting inflammation persistent even after 9 days of recovery and presented a more consistent microscopic pattern than the 0.25mg/ml DSS challenge. However, independent of the trigger, the inflammation response of the broilers showed a similar pattern and the intestinal inflammation progressed from the cranial segments of the small intestine to the caudal areas through time (**Figure 5**).

### Biomarkers

Calprotectin, lipocalin and LPS were analyzed in the serum of broilers but the only serum biomarker that showed a statistical difference in the challenged groups was calprotectin (CALP) (**Table 6**). The concentration of CALP in the blood was higher in the presence of intestinal inflammation (P-value = 0.0365) at 14 days of age. Calprotectin concentration in the serum was 135% higher in animals from the 35DSS group when compared with control birds. However, after the recovery period (at 22-d) of the first DSS cycle, the difference in calprotectin concentration of both groups disappeared. The rice bran presented a CALP elevation of 60.9% but it was not sufficient to be different than the CNT at 14-d. In addition, calprotectin concentration in the serum might be age-dependent. A 10-fold increase of calprotectin concentration in the serum was found in all treatments between days 14 and 22 (**Table 6**). The serum concentrations of lipocalin and LPS were not statistically different among the treatments at any of the timepoints measured (**Table 7** and **Supplemental Data-Table 7**, respectively).

**Table 6:** Calprotectin concentration in the serum of broilers submitted to different intestinal challenges at 14 and 22 days of age.

Treatment	14 days		22 days	
	Calprotectin (ng/ml)	± S.E.M.	Calprotectin (ng/ml)	± S.E.M.
Control	16.86 ^b^	1.74	221.34	36.61
25DSS	21.14 ^ab^	2.86	188.49	19.33
35DSS	39.68 ^a^*	8.29	232.47	21.17
NSP	27.13 ^ab^	4.81	203.79	18.83
**P-value**	0.0365		0.6562	

* Treatments differ from Control by Dunnet’s test (P-value<0.05).

^abc^ Different superscript letters indicate significant difference with Tukey test in the same column.

n= 6 animals/treatment; 2 animals/pen.

DSS: Dextran sodium sulfate; NSP: non-starch polyssacharide

The broilers challenged with DSS received 0.25mg/ml (25DSS) or 0.35mg/ml (35DSS) of DSS via oral gavage everyday from 9 to 14-d and 23 to 27-d; birds in the NSP treatment received a diet with 30% of rice bran during the whole experiment, and animals in the control group were not submitted to any challenge.

**Table 7:** Lipocalin concentration in the serum of broilers submitted to different intestinal challenges at 14, 22 and 28 days of age.

Treatment	14 days Lipocalin (ng/ml)	± S.E.M.	22 days Lipocalin (ng/ml)	± S.E.M.	28 days Lipocalin (ng/ml)	± S.E.M.
Control	85.60	3.66	90.06	7.83	50.21	6.28
25DSS	95.49	3.39	93.53	8.58	53.85	3.99
35DSS	86.67	4.70	94.85	7.83	48.8	5.46
NSP	86.86	2.97	84.72	7.83	60.36	4.64
**P-value**	0.2463		0.8061		0.4188	

n= 6 animals/treatment; 2 animals/pen.

DSS: Dextran sodium sulfate; NSP: non-starch polyssacharide.

The broilers challenged with DSS received 0.25mg/ml (25DSS) or 0.35mg/ml (35DSS) of DSS *via* oral gavage everyday from 9 to 14-d and 23 to 27-d; birds in the NSP treatment received a diet with 30% of rice bran during the whole experiment, and animals in the control group were not submitted to any challenge.

Among the analyzed fecal biomarkers, calprotectin and lipocalin showed statistical differences among treatments at day 28 (P-value = 0.0395 and P-value=0.0075, respectively; **Table 8**). After the second cycle of DSS, the 35DSS-treated birds showed an increase in fecal calprotectin concentration when compared to the control by 24.99%. DSS (25DSS) and NSP treatments had a numerical increase in fecal calprotectin concentration but no statistical difference compared to the control. Broilers fed the high NSP diet also showed a statistical increase in fecal lipocalin concentration (27.09%) (P-value = 0.0075). However, by 36 days of age, no differences were observed in either fecal calprotectin or lipocalin concentrations, suggesting there is an optimal time to analyze the markers or that the numbers of samples were not enough to detect differences among treatments in older birds. Interestingly, fecal HIF-1α presented a tendency (P-value=0.062) at 36 days of age with 35DSS showing the highest concentration. No differences were observed in the concentration of fecal S100 at any day analyzed or fecal ovotransferrin at 28-d (**Table 8** and **Supplemental Data Table 8**, respectively).

**Table 8:** Biomarker concentration on excreta of broilers submitted to different intestinal challenges at 28 and 36 days of age. The broilers challenged with DSS received 0.25mg/ml (25DSS) or 0.35mg/ml (35DSS) of DSS via oral gavage everyday from 9 to 14-d and 23 to 27-d; birds in the NSP treatment received a diet with 30% of rice bran during the whole experiment, and animals in the control group were not submitted to any challenge.

Collection day	Treatment	Calprotectin (ng/g)	± S.E.M.	Lipocalin (ng/g)	± S.E.M.	S100A (ng/g)	± S.E.M.	HIF-1α (pg/g)	± S.E.M.
28 Days	Control	5168.6 ^b^	238	3557.44^b^	264.8	118.7	5.49	1195.2	53.6
	25DSS	5705.1 ^ab^	246.9	3383.49^b^	186.1	103.9	3.6	1158.3	38.72
	35DSS	6460.7 ^a^*	88.53	3693.66^ab^	253.5	108.1	8.9	1063.9	104.6
	NSP	5986.8 ^ab^	413.7	4521.49^a^*	96.9	93.9	7.98	990.5	91.86
	**P-value**	0.0395		0.0075		0.1281		0.28	
**36 Days**		**Calprotectin**	** ± S.E.M.**	**Lipocalin (ng/g)**	** ± S.E.M.**	**S100A (ng/g)**	** ± S.E.M.**	**HIF-1α (pg/g)**	**± S.E.M.**
	Control	6176.4	488.3	1039.6	133.6	166.39	17.67	143.3	28.15
	25DSS	6321.7	725.4	1252.2	131.2	181.4	22.36	244.7	20.73
	35DSS	6171.7	485.6	1156	154.3	160.6	16.32	249.6	24.93
	NSP	6365.7	480.4	1360.6	141.7	135.7	8.64	225	39.6
	**P-value**	0.992		0.4364		0.3174		0.0627	

*Treatments differ from Control by Dunnet’s test (P-value < 0.05)..

^abc^ Different superscript letters indicate significant difference with Tukey test in the same column.

n = 6 animals/treatment; 2 animals/pen.

DSS, Dextran sodium sulfate; NSP, non-starch polyssacharide.

## Discussion

### Inflammation Model: DSS and NSP

The present data revealed that the intestinal mucosal reaction (i.e. inflammation) to stressors followed a spatial and temporal pattern in broilers, from the duodenum to the ileum from 14 to 36 days post-hatch (**Figure 5**). The histological assessment indicated that the duodenum and jejunum were affected earlier than the ileum. Thus, it seems that the lower part of the gut was affected only after the upper gut was compromised in terms of intestinal health (**Figures 2**–**4**). Duodenum and jejunum had signs of injury at the first necropsy (14-d). However, indications of acute inflammation, such as the presence of inflammatory cells in the lamina propria and epithelium were observed in the ileum from day 22 and were maintained until the end of the experiment in 35DSS and NSP groups. Evidence of a higher upper gut sensitivity has already been described in broilers exposed to heat stress with greater histo-morphological and morphometric alteration observed in the duodenum and jejunum (37). Therefore, the stronger inflammatory response of the upper gut might be not related to the stressor source, since it happened with NSP rich diet, chemical (DSS) and even heat challenge. However, the spatial characteristics of intestinal inflammation verified in the present study contrasted with findings in the murine model. Previous research showed that the distal colon of mice is more affected by DSS challenge than its proximal colon (38–41).

Overall, NSP exhibited a more consistent pattern of inflammation during the trial although it was milder when compared with the DSS-treated birds (**Figures 2**, **3**). NSP negatively affected intestinal health, as seen by the significantly higher macroscopic ISI score as well as the microscopic lesion scores (**Table 5** and **Figures 2**, **3**).

DSS challenge promoted higher levels of micro-ISI scores in the duodenum than control (P<0.001) throughout the entire experiment (**Figure 2**). And at the last day of sampling (36-d), the 35DSS group presented the worst (highest) ISI scores throughout the small intestine. The higher ISI scores were mainly due to the high inflammatory cell infiltration in the lamina propria and epithelium. DSS groups showed a consistent increase in the number of goblet cells through the end of the trial (28 and 36-d). The DSS model proposed in this paper was adapted to chickens based on a mice model for chronic inflammation (23). These authors observed that mice recovered faster from the first DSS cycle (9-d) but, after the second round of chemical oral gavage 9-d was not enough to have a full recovery from the inflammation caused by DSS. Our study demonstrated that broilers exposed to a similar protocol presented a major intestinal inflammation in the second DSS cycle as was observed in the murine model (23). However, for broilers, 9 days was not sufficient for full recovery from the first challenge cycle. Since chickens appear to be more sensitive to oral DSS (10), the current observation is not surprising. Moreover, in mice the inflammatory response after the second recovery period is different from the response after de first recovery. After two cycles of DSS and the full recovery period, mice presented a chronic inflammation identified by the infiltration of mononuclear cells, and imbalance of pro and anti-inflammatory response. Although in the present study, we did observe a prolonged inflammatory response in the three intestinal segments evaluated even after the second recovery, we did not categorize and quantify the inflammatory cells and cytokines present in the intestine at each time point.

In the current experiment, all the challenged groups presented a high level of enterocyte proliferation and inflammatory cell infiltration in the epithelium of the small intestine at 28 days. Sanches and colleagues (8) observed that during inflammation the avian intestine responds to the lesions in the lamina propria mostly with a strong proliferation of enterocytes that further stimulates the inflammatory response since these new enterocytes are not mature. They also described a microscopic proliferative enteritis in broilers with *Eimeria spp*. and *Clostridium perfringens*, confirming this pattern.

Furthermore, the current data suggest that histologic methodologies are superior to the evaluation of gross lesions for small intestine inflammation in research settings. Other than its precision and ability to provide details, a histologic evaluation may be crucial to detect Microscopic Enteritis (ME). Human pathology uses ME as a classification for an inflammatory condition of the small intestine which presents microscopic or sub-microscopic abnormalities but not gross lesions (42). The histological observations in ME include preserved villous structure, crypt hyperplasia (deeper crypt), infiltration of small lymphocytes in the epithelium, sub-microscopic abnormalities, increase in plasma cells and inflammatory cells in the lamina propria also may be present. Even if the lesions appear minor, ME is associated with gastrointestinal symptoms, and malabsorption (43). Microscopic enteritis was already identified in broilers through microscopic ISI evaluation (8), and Belote et al. (33) have shown that the increase of inflammatory cells in the epithelium has a negative impact on animal performance by reducing growth performance.

### Biomarkers

The poultry industry is constantly looking for tools to measure gut health in order to evaluate flocks and decide when to use feed additives or medications. Moreover, these tools should be: (1) able to provide a rapid answer to allow intervention during the production period; (2) minimally invasive; and (3) easy to collect and store the required samples. Several ways to identify gut inflammation and/or gut health are available for research settings; however, most require tissue sampling and euthanasia of animals. Thus, blood and excreta were chosen in the current experiment to fulfill the 2^nd^ and 3^rd^ requirements. Moreover, ELISA assays were selected for their relative simplicity in methodology, low requirement in lab equipment, quick generation of results, high specificity and easy commercial access. It is believed that the relevant findings from this research will facilitate further research in different labs including widespread industry application. ELISA assays offer an additional advantage over molecular methods (i.e. mRNA gene expression) in the sense that the targeted biomarker refers to finally expressed (protein) levels, which is not the case in the latter.

From the biomarkers evaluated in the current experiment, calprotectin was observed to be the most promising. Calprotectin, also known as S100A8/S100A9 heterocomplex, is a protein involved in the innate immune response to infection. It activates pro-inflammatory signaling pathways and has chemokine and bacteriostatic activity (44–47). Avian and reptile species express a protein homologue to calprotectin, named MRP126. This protein has already been found in abundance in chicken heterophils and in lower quantities in macrophages (48, 49). Recently, the chicken MRP126 has been biophysically described by Bozzi and Nolan with important observation of its metal binding (Ca^+2^-dependent Zn^+2^ sequestration) and antimicrobial property activities (50). Moreover, recombinant chicken MRP126 had been shown to stimulate chicken TLR4 to activate NF-κB (51). Therefore, it appears that the cytokine-like and bacteriostatic activity of MRP126 protein was maintained during evolution. A previous study has shown that neutrophils release calprotectin with activation or death and monocytes release the protein after adhesion (52). Thus, calprotectin can be detected in fluids of inflamed tissue, such as serum, urine, cerebrospinal fluid and feces (53). Sekelova and collaborators (49) observed that the abundance of MRP126 protein increase in avian macrophages after intravenous infection with *Salmonella* Enteritidis. Therefore, the increased calprotectin from blood and feces found in the current study might be correlated to the immune response of the challenged groups. The microscopic ISI scores and the histologic alterations indicate the presence of an inflammatory response in the intestine, specifically identified by the increase in inflammatory cells in epithelium and lamina propria, and congestion. Although fluctuations were observed throughout the experimental period, calprotectin was increased only right after the DSS cycles. Two factors can play a role in this finding: first, calprotectin is more abundant in heterophils, and second, the number of animals sampled may not be sufficient to detect small variations in the concentration of proteins after the recovery period. Heterophils are the first immune cells to respond and migrate to a site of infection (54) and are found in great quantity in acute inflammation. However, with the evolution of acute to chronic inflammation more mononuclear cells are identified with macrophages being the predominant cell type in chronically inflamed tissues (55). Thus, the change in cell type predominance at the moment of the sampling may have influenced the calprotectin concentration, suggesting the protein as an indicator of more robust inflammation. Calprotectin may be a promising biomarker for inflammation in broiler measured both in serum and excreta.

The ideal biomarker should also detect inflammation consistently in different models, such as physiological and nutritional models (13); therefore, in the current study, we also analyzed biomarkers previously described in the literature such as LPS, ovotransferrin and lipocalin-2. Lipocalin 2 is an acute phase protein highly produced by the liver (56) and neutrophils, although the majority of LCN2 found in the intestine is produced by epithelial cells which mostly secrete the protein into the lumen (57). Similar to calprotectin, lipocalin inhibits bacterial growth by sequestering nutrients from the bacteria, such as iron (56), and it also is involved in apoptosis (58). Chassaing and collaborators (59) reported that fecal LCN-2 is a sensitive marker capable of monitoring low-grade (sub-clinical) and robust intestinal inflammation in mice since the protein showed responsiveness to mucosal healing and high stability in the feces. The authors observed an increase in fecal LCN-2 after challenging mice with different dosages of DSS in the drinking water, and when the challenge was discontinued, LCN-2 dramatically decreased its concentration. However, they observed that lipocalin concentration in the serum was less responsive to the DSS challenge, being a better detector of robust intestinal inflammation (caused by high doses of DSS). Similarly, these current results show that LCN-2 appears to be a better marker of low-grade inflammation in broilers in the feces than in the serum. In the present trial, broilers fed for 28 days with the NSP showed higher fecal lipocalin at 28 days, compared to the control group. However, with the healing of the upper gut after the first recovery period, LCN-2 returned to basal levels, indicating LCN-2 as a potential fecal biomarker for the health status of upper gut segments. Previously, it has been reported that fecal lipocalin in chickens (13) had a tendency (P=0.086) to increase in a dexamethasone model of intestinal inflammation. However, the lipocalin differences probably was less prominent due to the control treatment used which had a diet with high inclusion of wheat (65.2%). This diet may have negatively impacted the gut due to its high soluble fiber content promoting inflammation and reducing the differences between control and other treatments in the study. Therefore, the current data sustain previous findings in the literature and supports the value of lipocalin as a biomarker for intestinal inflammation in chickens.

The LPS present in the serum was quantified to detect bacterial translocation from the intestinal lumen to blood, thus evaluating intestinal barrier function. However, no difference between challenge groups and control was found at 14, 28 or 36 days. The measuring of serum LPS in chickens is controversial in the literature and it seems to vary according to the intensity of the challenge to which birds are exposed (60–62). Ovotransferrin, an acute phase protein mainly produced during inflammation, has been suggested as a fecal biomarker of intestinal inflammation due to its increase in excreta of broilers facing necrotic enteritis and coccidiosis (25), or diet with 52% of rye (13). However, in our study, no increase of fecal ovotransferrin was detected in the groups suffering intestinal inflammation, which might occur due to the sub-clinical inflammation observed in the current study compared to the acute and more robust inflammation produced by the other models in the literature.

Lastly, HIF-1α is a transcription factor that regulates genes involved in inflammation and cell death (63). HIF-1α has been shown to be an important hypoxia-elicited barrier protection through nonclassical barrier function (non-tight junctions related) (64). Moreover, the HIF-1α pathway can be activated by NF-kB, thus initiating inflammation and vice versa (64, 65). Gene expression of HIF-1α has been measured in broilers facing intestinal inflammation due to heat stress and an up-regulation of the HIF-1α gene was detected in the ileum, but not jejunum (66). Our results followed a similar response, since the HIF-1α concentration presents a tendency to be increased in the feces of broilers with lower ileum health status (P = 0.0627). Likewise, He and collaborators (67) only observed intestinal upregulation of HIF-1α expression after a chronic heat stress exposure (for 14 days), but not after 7 days of thermal challenge. Although HIF-1α levels need to be further quantified in the excreta of broilers, the HIF-1α seems to be a potential fecal biomarker for chronic inflammation or ileal health status. Further experiments are underway to confirm the use of HIF-1α as a potential inflammatory marker.

The ideal biomarker has been described with several characteristics: minimally-invasive, specific, precocious, sensitive, robust, consistent among different challenge models and responsive to tissue healing (13, 59, 68). However, an important point that is usually missed is the practicality of sampling and processing, and the time to produce results. Although, authors advocate that fecal biomarkers should be more accurate in determining gastrointestinal inflammation and gut barrier (69), our data demonstrated that serum biomarker (calprotectin) presented bigger differences (135%) compared to fecal biomarkers (CALP and LCN-2) (~25% increase). A biomarker with a broader range would permit a better distinction among good gut health, low-grade and robust inflammation. The difference between the biomarkers response in serum and feces can be physiological, due to age and may be associated with the difficulty of fecal processing, variation and representativeness. The presence of proteases in the intestinal content can diminish some proteins with biomarker potential even before the excretion. Moreover, fecal samples are vulnerable to protein degradation and usually are flash frozen right after sampling in liquid nitrogen and stored at -80°C (13, 25). This methodology might impair successful collections in the field. In our experiment, we observed easy handling and processing of the blood samples, minimal equipment needed, combined with the advantage of monitoring individual birds at the same time on necropsy days. Therefore, to benefit the research performed in industry settings, there should be a focus not solely on feces but also on blood biomarkers, as well as to further evaluate biomarkers stability in the samples throughout different experimental situations.

## Conclusion

Both NSP diet and two cycles of 0.35mg/ml of DSS models produced an inflammatory response in the intestine without compromising other organs or producing clinical signs. Therefore, this study presents feasible models of low-grade chronic inflammation although the judgment between the models should be made based on the time response desired and the practicality. The high NSP diet is a nutritional model which is easy to replicate and should be used if an early intestinal inflammation is preferred (up to 28d). On the other hand, cyclic DSS challenges is a chemical model which continues producing inflammation later in the broiler’s life (up to 36d). Interestingly, the current study demonstrated that the chicken intestinal inflammation evolves in a spatial and temporal pattern, shown by the lower small intestine being affected later than the upper parts. After a challenge, the duodenum and jejunum were affected earlier, followed by the ileum which was compromised only after 4-wk of the life span of the chicken. Moreover, this physiological response might not vary with the stressor’s characteristic since it was consistent between a nutritional-continuous challenge and a cyclic-chemical model.

Lastly, we described promising biomarkers for low-grade intestinal inflammation in the current paper such as calprotectin, lipocalin and HIF-1α. Calprotectin, a novel biomarker for chickens, was successfully detected in response to inflammation from serum and feces, while HIF-1α and lipocalin may be potential fecal markers. To the best of our knowledge, this is the first time that calprotectin has been described as a biomarker of intestinal health in broiler chickens. Even more promising, is the fact that it can be easily analyzed in the blood and be correlated with intestinal health, which may reduce the variation between birds, be less time-consuming, and reduce the risk of degradation during sample processing.

## Data Availability Statement

The original contributions presented in the study are included in the article/**Supplementary Material**. Further inquiries can be directed to the corresponding authors.

## Ethics Statement

The animal study was reviewed and approved by United States Department of Agriculture Animal Care and Use Committee (USDA ACUC #2019012).

## Author Contributions

GD: Conceptualization, Conducted experiment and laboratory analysis, Writing, and Editing. BB: Laboratory analysis, Writing. AL: Assistant in the experiment, Reviewing, Editing. CE: Assistant in the experiment, Reviewing. CB: Assistant in the experiment, Reviewing. MS: Reviewing. ES: Data analysis and interpretation, Reviewing and editing. YF: Reviewing, Supervision. CG*: Data analysis and interpretation, Reviewing. MK*: Conceptualization, Data analysis and interpretation, Reviewing, Supervision. *Share last authorship. All authors contributed to the article and approved the submitted version.

## Conflict of Interest

The authors declare that the research was conducted in the absence of any commercial or financial relationships that could be construed as a potential conflict of interest.
