# Supplementary material for: Novel Models for Chronic Intestinal Inflammation in Chickens: Intestinal Inflammation Pattern and Biomarkers
Source: Front Immunol. 2021 May 12;12:676628. doi: 10.3389/fimmu.2021.676628 (PMC8158159; doi:10.3389/fimmu.2021.676628)
Supplement: Supplementary file 1 [file DataSheet_1.docx]

**Supplemental Data**

***Macroscopic I See Inside methodology***

**Table 1.** Characteristics evaluated and their impact factors (IF) used for calculation of the macroscopic I See Inside (ISI). Each characteristic was scored based in the extend of the lesion being: (0) absence of lesion, (1) up to 25% of the organ affected, (2) 25-50% of the organ affected, (3) more than 50% of the organ affected. Then scores were multiplied by their respective impact factors and the sum of all score*IF of an animal resulted in its macroscopic ISI.

| Organ | Characteristic evaluated | Impact factor |
| --- | --- | --- |
| Liver | Red color (congestion) | 2 |
|  | Yellow and hypertrophic | 2 |
|  | Yellow and hypotrophic | 3 |
| Yolk | Persistance | 1 |
| Proventriculus | Inflammation | 1 |
| Gizzard | Erosion | 1 |
| Pancreas | Hypertrophic | 2 |
|  | Hypotrophic | 2 |
| Duodenum | Inflammation process at serosa or mucosa layer | 1 |
|  | Cell debris and thick mucus at mucosa | 2 |
|  | *Eimeria acervulina* lesion (0-4) | 2 |
| Jejunum | Necrosis | 3 |
|  | Inflammation process at serosa or mucosa layer | 1 |
|  | Cell debris and thick mucus at mucosa | 2 |
|  | Decrease muscular tonus (thin intestine wall) | 2 |
|  | *Eimeria maxima* lesion (0-4) | 3 |
| Ileum | Inflammation process at serosa or mucosa layer | 1 |
|  | Cell debris and thick mucus at mucosa | 2 |
|  | Undigested food and/or gas presence | 1 |
| Ceacum | Inflammation process at mucosa | 2 |
|  | Gas presence | 1 |
|  | *Eimeria tenella* lesion (0-4) | 2 |

**Table 2.** Broiler mortality (%) in the entire experimental period and feed intake (FI) of 1 to 21 days and 1 to 36 days of broilers submitted to different intestinal challenges. The broilers challenged with DSS received 0.25mg/ml (25DSS) or 0.35mg/ml (35DSS) of DSS via oral gavage everyday from 9 to 14-d and 23 to 27-d; birds in the NSP treatment received a diet with 30% of rice bran during the whole experiment, and animals in the control group were not submitted to any challenge.

| Treatment | FI (kg)  1-21 days | FI (kg)  1-36 days | Mortality (%)  1-36 days |
| --- | --- | --- | --- |
| Control | 1.037 | 2.442 | 0 |
| 25DSS | 1.094 | 2.573 | 2.22 |
| 35DSS | 1.049 | 2.422 | 2.22 |
| NSP | 1.019 | 2.312 | 2.22 |
| SEM^1^ | 0.04337 | 0.10545 | - |
| P-value | 0.6663 | 0.4269 | 0.7904 |

^1^ Pooled standard error of the mean

**Table 3.** Detailed histologic alteration on duodenum, jejunum and ileum of broilers submitted to different intestinal challenges at 14 of age. The broilers challenged with DSS received 0.25mg/ml (25DSS) or 0.35mg/ml (35DSS) of DSS via oral gavage everyday from 9 to 14-d and 23 to 27-d; birds in the NSP treatment received a diet with 30% of rice bran during the whole experiment, and animals in the control group were not submitted to any challenge.

|  | **Treatment** | **Lamina propria thickness** | **Epithelial thickness** | **Proliferation of enterocytes** | **Inflammatory cell epithelium** | **Inflammatory cell lamina propria** | **Increase of goblet cells** | **Congestion** |  |
| --- | --- | --- | --- | --- | --- | --- | --- | --- | --- |
| **Duodenum** | **Control** | 0.93^ab^ | 1.08^ab^ | 0.67 | 0.43^b^ | 0.27^b^ | 1.30^b^ | 0.17 |  |
|  | **25DSS** | 1.27^a^ | 1.22^a^ | 0.53 | 0.87^a^ | 0.80^a^ | 2.17^a^ | 0.20 |  |
|  | **35DSS** | 1.20^a^ | 0.95^b^ | 0.57 | 0.80^a^ | 0.57^a^ | 1.60^b^ | 0.43 |  |
|  | **NSP** | 0.77^b^ | 0.83^b^ | 0.43 | 0.87^a^ | 0.87^a^ | 0.87^c^ | 0.47 |  |
| **Jejunum** | **Control** | 0.80 | 0.93 | 0.56^d^ | 0.53^b^ | 0.50 | 1.50 | 0.36^a^ |  |
|  | **25DSS** | 1.36 | 1.20 | 0.96^bc^ | 0.90^a^ | 0.76 | 1.90 | 0.46^a^ |  |
|  | **35DSS** | 1.26 | 1.13 | 0.85^c^ | 0.86^a^ | 0.76 | 1.73 | 0.46^a^ |  |
|  | **NSP** | 1.13 | 1.26 | 1.08^ab^ | 0.91^a^ | 0.80 | 1.63 | 0.06^b^ |  |
| **Ileum** | **Control** | 0.60^b^ | 0.80 | 0.60 | 0.53 | 0.23 | 1.27 | 0 |  |
|  | **25DSS** | 1.00^ab^ | 0.72 | 0.65 | 0.73 | 0.70 | 0.77 | 0 |  |
|  | **35DSS** | 1.23^a^ | 0.92 | 0.83 | 0.77 | 0.50 | 1.20 | 0 |  |
|  | **NSP** | 1.43^a^ | 0.95 | 0.70 | 0.78 | 0.77 | 0.80 | 0 |  |
| **Duodenum** | SEM^1^ | 0.152 | 0.096 | 0.079 | 0.082 | 0.128 | 0.169 | 0.118 |  |
|  | P-value | 0.0156 | 0.0279 | 0.2066 | 0.0002 | 0.0077 | <.0001 | 0.1789 |  |
| **Jejunum** | SEM^1^ | 0.159 | 0.089 | 0.081 | 0.078 | 0.132 | 0.197 | 0.101 |  |
|  | P-value | 0.1005 | 0.0609 | <.0001 | 0.0008 | 0.3497 | 0.4998 | 0.0159 |  |
| **Ileum** | SEM^1^ | 0.171 | 0.089 | 0.084 | 0.085 | 0.144 | 0.164 | 0 |  |
|  | P-value | 0.0045 | 0.2196 | 0.285 | 0.2438 | 0.0731 | 0.0561 | 1 |  |

^abc^ Different superscript letters indicate significant difference with Tukey test in the same column.

^1^ Pooled standard error of the mean

**Table 4.** Detailed histologic alteration on duodenum, jejunum and ileum of broilers submitted to different intestinal challenges at 22 of age. The broilers challenged with DSS received 0.25mg/ml (25DSS) or 0.35mg/ml (35DSS) of DSS via oral gavage everyday from 9 to 14-d and 23 to 27-d; birds in the NSP treatment received a diet with 30% of rice bran during the whole experiment, and animals in the control group were not submitted to any challenge.

|  | **Treatment** | **Lamina propria thickness** | **Epithelial thickness** | **Proliferation of enterocytes** | **Inflammatory cell epithelium** | **Inflammatory cell lamina propria** | **Increase of goblet cells** | **Congestion** |
| --- | --- | --- | --- | --- | --- | --- | --- | --- |
| Duodenum | **Control** | 0.67 | 0.78 | 0.60 | 0.42^b^ | 0.37 | 0.73^b^ | 0.23 |
|  | **25DSS** | 0.80 | 1.00 | 0.68 | 0.65^a^ | 0.67 | 1.13^b^ | 0.23 |
|  | **35DSS** | 0.87 | 1.10 | 0.73 | 0.70^a^ | 0.73 | 1.53^a^ | 0.23 |
|  | **NSP** | 0.93 | 1.10 | 0.85 | 0.73^a^ | 0.60 | 0.97^b^ | 0.07 |
| Jejunum | **Control** | 0.77^b^ | 0.77^c^ | 0.50^b^ | 0.87^c^ | 0.50^b^ | 1.07^c^ | 0.00^b^ |
|  | **25DSS** | 1.37^a^ | 1.65^a^ | 1.23^a^ | 1.95^a^ | 1.67^a^ | 2.90^a^ | 0.47^a^ |
|  | **35DSS** | 0.80^b^ | 1.28^b^ | 1.02^a^ | 1.32^b^ | 0.73^b^ | 2.47^ab^ | 0.20^a^ |
|  | **NSP** | 0.80^b^ | 1.45^ab^ | 1.07^a^ | 1.58^b^ | 0.67^b^ | 2.07^b^ | 0.20^a^ |
| Ileum | **Control** | 1.10^b^ | 1.32^a^ | 0.87 | 1.08 | 0.73^b^ | 1.47^a^ | 0.23 |
|  | **25DSS** | 1.47^b^ | 1.25^a^ | 1.02 | 1.22 | 1.00^b^ | 1.77^a^ | 0.27 |
|  | **35DSS** | 2.40^a^ | 0.82^b^ | 0.80 | 1.07 | 2.20^a^ | 0.83^b^ | 0.10 |
|  | **NSP** | 2.30^a^ | 1.05^b^ | 0.97 | 1.13 | 2.10^a^ | 0.73^b^ | 0.17 |
| Duodenum | SEM^1^ | 0.148 | 0.085 | 0.082 | 0.088 | 0.135 | 0.160 | 0.080 |
|  | P-value | 0.6825 | 0.0221 | 0.1159 | 0.0343 | 0.3141 | 0.0027 | 0.3369 |
| Jejunum | SEM^1^ | 0.148 | 0.106 | 0.092 | 0.094 | 0.143 | 0.210 | 0.100 |
|  | P-value | 0.0182 | <.0001 | <.0001 | <.0001 | <.0001 | <.0001 | 0.0232 |
| Ileum | SEM^1^ | 0.161 | 0.094 | 0.090 | 0.086 | 0.182 | 0.196 | 0.080 |
|  | P-value | <.0001 | 0.0008 | 0.2619 | 0.6845 | <.0001 | 0.0011 | 0.4596 |

^abc^ Different superscript letters indicate significant difference with Tukey test in the same column.

^1^ Pooled standard error of the mean

**Table 5.** Detailed histologic alteration on duodenum, jejunum and ileum of broilers submitted to different intestinal challenges at 28 of age. The broilers challenged with DSS received 0.25mg/ml (25DSS) or 0.35mg/ml (35DSS) of DSS via oral gavage everyday from 9 to 14-d and 23 to 27-d; birds in the NSP treatment received a diet with 30% of rice bran during the whole experiment, and animals in the control group were not submitted to any challenge.

|  | **Treatment** | **Lamina propria thickness** | **Epithelial thickness** | **Proliferation of enterocytes** | **Inflammatory cell epithelium** | **Inflammatory cell lamina propria** | **Increase of goblet cells** | **Congestion** |
| --- | --- | --- | --- | --- | --- | --- | --- | --- |
| Duodenum | **Control** | 0.53^b^ | 0.62^c^ | 0.42^c^ | 0.62^b^ | 0.27^c^ | 0.73^c^ | 0.47 |
|  | **25DSS** | 1.07^a^ | 1.30^a^ | 1.03^a^ | 1.18^a^ | 0.77^ab^ | 1.93^a^ | 0.77 |
|  | **35DSS** | 1.27^a^ | 1.25^a^ | 0.93^ab^ | 1.12^a^ | 1.07^a^ | 1.87^a^ | 0.60 |
|  | **NSP** | 0.67^b^ | 0.95^b^ | 0.72^bc^ | 1.13^a^ | 0.63^b^ | 1.23^b^ | 0.63 |
| Jejunum | **Control** | 0.53^b^ | 0.42^b^ | 0.35^b^ | 0.52^b^ | 0.47^b^ | 0.47^c^ | 0.37^b^ |
|  | **25DSS** | 1.13^a^ | 1.20^a^ | 1.00^a^ | 1.22^a^ | 1.03^a^ | 1.97^a^ | 0.83^a^ |
|  | **35DSS** | 0.77^ab^ | 1.12^a^ | 0.90^a^ | 1.25^a^ | 0.83^ab^ | 1.40^b^ | 0.13^b^ |
|  | **NSP** | 1.07^a^ | 0.92^a^ | 0.80^a^ | 1.12^a^ | 0.70^ab^ | 1.00^b^ | 0.37^b^ |
| Ileum | **Control** | 1.03^c^ | 0.62^b^ | 0.53^b^ | 0.37^b^ | 0.40^b^ | 0.73 | 0.10 |
|  | **25DSS** | 1.97^ab^ | 1.12^a^ | 0.95^a^ | 1.23^a^ | 1.47^a^ | 1.13 | 0.10 |
|  | **35DSS** | 2.27^a^ | 1.12^a^ | 1.10^a^ | 1.12^a^ | 1.17^a^ | 0.87 | 0.17 |
|  | **NSP** | 1.87^b^ | 1.02^a^ | 0.97^a^ | 1.15^a^ | 1.40^a^ | 0.77 | 0.00 |
| Duodenum | SEM^1^ | 0.141 | 0.105 | 0.100 | 0.104 | 0.131 | 0.182 | 0.141 |
|  | P-value | 0.0007 | <.0001 | <.0001 | 0.0002 | 0.0007 | <.0001 | 0.4055 |
| Jejunum | SEM^1^ | 0.134 | 0.098 | 0.097 | 0.097 | 0.136 | 0.190 | 0.117 |
|  | P-value | 0.0035 | <.0001 | <.0001 | <.0001 | 0.0178 | <.0001 | 0.0021 |
| Ileum | SEM^1^ | 0.147 | 0.081 | 0.083 | 0.079 | 0.149 | 0.148 | 0.059 |
|  | P-value | <.0001 | <.0001 | <.0001 | <.0001 | <.0001 | 0.3023 | 0.1485 |

^abc^ Different superscript letters indicate significant difference with Tukey test in the same column.

^1^ Pooled standard error of the mean

**Table 6.** Detailed histologic alteration on duodenum, jejunum and ileum of broilers submitted to different intestinal challenges at 36 of age. The broilers challenged with DSS received 0.25mg/ml (25DSS) or 0.35mg/ml (35DSS) of DSS via oral gavage everyday from 9 to 14-d and 23 to 27-d; birds in the NSP treatment received a diet with 30% of rice bran during the whole experiment, and animals in the control group were not submitted to any challenge.

|  | **Treatments** | **Lamina propria thickness** | **Epithelial thickness** | **Proliferation of enterocytes** | **Inflammatory cell epithelium** | **Inflammatory cell lamina propria** | **Increase of goblet cells** | **Congestion** |
| --- | --- | --- | --- | --- | --- | --- | --- | --- |
| Duodenum | **Control** | 0.73 | 1.17 | 0.58 | 1.02^b^ | 0.63^b^ | 1.67^b^ | 0.30^b^ |
|  | **25DSS** | 0.97 | 1.20 | 0.87 | 1.18^ab^ | 0.90^b^ | 2.33^a^ | 0.87^a^ |
|  | **35DSS** | 1.23 | 1.17 | 0.83 | 1.45^a^ | 1.30^a^ | 2.17^ab^ | 0.37^b^ |
|  | **NSP** | 1.10 | 1.02 | 0.78 | 1.05^b^ | 0.90^b^ | 1.57^b^ | 0.27^b^ |
| Jejunum | **Control** | 1.00^b^ | 0.52^c^ | 0.38^c^ | 0.80^c^ | 1.17^a^ | 0.63^b^ | 0.30 |
|  | **25DSS** | 0.87^b^ | 0.87^b^ | 0.65^b^ | 1.15^ab^ | 0.87^b^ | 1.03^b^ | 0.63 |
|  | **35DSS** | 1.63^a^ | 1.17^a^ | 0.93^a^ | 1.30^a^ | 1.60^a^ | 1.63^a^ | 0.33 |
|  | **NSP** | 1.00^b^ | 0.80^b^ | 0.70^ab^ | 1.03^b^ | 0.60^b^ | 0.60^b^ | 0.30 |
| Ileum | **Control** | 1.03^b^ | 0.83^b^ | 0.65 | 0.75^b^ | 0.57^c^ | 0.97^b^ | 0.10 |
|  | **25DSS** | 1.57^b^ | 0.72^b^ | 0.75 | 0.92^b^ | 1.10^b^ | 0.63^b^ | 0.07 |
|  | **35DSS** | 2.17^a^ | 1.02^a^ | 0.85 | 1.20^a^ | 1.77^a^ | 1.47^a^ | 0.30 |
|  | **NSP** | 1.43^b^ | 1.18^a^ | 0.98 | 1.20^a^ | 0.90^b^ | 0.93^b^ | 0.10 |
| Duodenum | SEM^1^ | 0.152 | 0.106 | 0.099 | 0.096 | 0.151 | 0.191 | 0.119 |
|  | P-value | 0.0822 | 0.5756 | 0.2545 | 0.0159 | 0.0068 | 0.0205 | 0.0022 |
| Jejunum | SEM^1^ | 0.142 | 0.097 | 0.089 | 0.085 | 0.148 | 0.168 | 0.110 |
|  | P-value | 0.0010 | 0.0001 | 0.0003 | 0.0006 | 0.0001 | <.0001 | 0.1336 |
| Ileum | SEM^1^ | 0.171 | 0.100 | 0.096 | 0.083 | 0.173 | 0.172 | 0.070 |
|  | P-value | 0.0003 | 0.0042 | 0.0535 | 0.0001 | 0.0001 | 0.0049 | 0.1136 |

^abc^ Different superscript letters indicate significant difference with Tukey test in the same column.^1^ Pooled standard error of the mean

**Table 7.** Serum liposaccharide (LPS) of broilers submitted to different intestinal challenges at 14, 28 and 36 days of age. The broilers challenged with DSS received 0.25mg/ml (25DSS) or 0.35mg/ml (35DSS) of DSS via oral gavage every daily from 9 to 14-d and 23 to 27-d; birds in the NSP treatment received a diet with 30% of rice bran during the whole experiment, and animals in the control group were not submitted to any challenge.

| Treatment | 14 days  LPS (ng/ml) | ±S.E.M. | 28 days  LPS (ng/ml) | ±S.E.M. | 36 days  LPS (ng/ml) | ±S.E.M. |
| --- | --- | --- | --- | --- | --- | --- |
| Control | 130.83 | 7.45 | 133.94 | 12.45 | 99.29 | 11.64 |
| 25DSS | 136.25 | 8.59 | 84.31 | 7.50 | 86.20 | 23.91 |
| 35DSS | 115.48 | 19.80 | 100.83 | 23.83 | 70.98 | 11.79 |
| NSP | 103.6 | 11.64 | 101.22 | 24.56 | 65.47 | 14.39 |
| P-value | 0.3199 |  | 0.3602 |  | 0.4149 |  |

**Table 8.** Concentration of Fecal ovotransferrin of broilers submitted to different intestinal challenges at 14 and 22 days of age. The broilers challenged with DSS received 0.25mg/ml (25DSS) or 0.35mg/ml (35DSS) of DSS via oral gavage daily from 9 to 14-d and 23 to 27-d; birds in the NSP treatment received a diet with 30% of rice bran during the whole experiment, and animals in the control group were not submitted to any challenge.

| Treatment | 28 days  Ovotransferrin (pg/g) | ±S.E.M. |
| --- | --- | --- |
| Control | 1008.76 | 86.948 |
| 25DSS | 961.93 | 97.211 |
| 35DSS | 948.89 | 86.948 |
| NSP | 1001.38 | 86.948 |
| P-value | 0.9526 |  |
